# Fluid Intelligence as a Mediator of the Relationship between Executive Control and Balanced Time Perspective

**DOI:** 10.3389/fpsyg.2016.01844

**Published:** 2016-11-22

**Authors:** Marcin Zajenkowski, Maciej Stolarski, Joanna Witowska, Oliwia Maciantowicz, Paweł Łowicki

**Affiliations:** Faculty of Psychology, University of WarsawWarsaw, Poland

**Keywords:** executive function, intelligence, fluid intelligence, time perspective, balanced time perspective

## Abstract

This study examined the cognitive foundations of the balanced time perspective (BTP) proposed by Zimbardo and Boyd (1999). Although BTP is defined as the mental ability to switch effectively between different temporal perspectives, its connection with cognitive functioning has not yet been established. We addressed this by exploring the relationships between time perspectives and both fluid intelligence (measured with Raven’s and Cattell’s tests) and executive control (Go/No-go and anti-saccade tasks). An investigation conducted among Polish adults (*N* = 233) revealed that more balanced TP profile was associated with higher fluid intelligence, and higher executive control. Moreover, we found that the relationship between executive control and BTP was completely mediated by fluid intelligence with the effect size (the ratio of the indirect effect to the total effect) of 0.75, which suggests that cognitive abilities play an important role in adoption of temporal balance. The findings have relevance to time perspective theory as they provide valuable insight into the mechanisms involved in assigning human experience to certain time frames.

## Introduction

The balanced time perspective (BTP) construct is a core feature of Zimbardo and Boyd’s (1999) time perspective theory. Time perspective (TP) is defined as an “often non-conscious process whereby the continual flow of personal and social experiences are assigned to temporal categories, or time frames, that help to give order, coherence, and meaning to those events” (Zimbardo and Boyd, 1999, p. 1271). TP includes social, cognitive, and emotional components and is frequently treated as a cognitive schema (Epel et al., 1999) or a cognitive process (Keough et al., 1999; Zimbardo and Boyd, 1999), which suggests that it may be related to other cognitive functions. Individuals usually develop a preference for one particular time frame (i.e., past, present, or future) and hence a relatively stable TP bias emerges (Boniwell and Zimbardo, 2004). Zimbardo and Boyd (1999) distinguished five TP dimensions empirically: Past-Positive, Past-Negative, Present-Fatalistic, Present-Hedonistic and Future. They also defined what they referred to as BTP - a specific, adaptive constellation of the five abovementioned dimensions. BTP can be defined statically as the combination of a high Past-Positive score, moderately high Present-Hedonistic and Future scores and low Past-Negative and Present-Fatalistic scores. However, it can also be considered as a dynamic process, i.e., the ability to switch between particular temporal perspectives according to the context (Boniwell and Zimbardo, 2004).

There is evidence that a BTP is associated with numerous positive outcomes, including positive mood (Stolarski et al., 2014), life satisfaction, happiness, psychological need satisfaction, self-determination (Zhang et al., 2013), emotional intelligence (Stolarski et al., 2011) and mindfulness (Stolarski et al., 2016). It therefore seems reasonable to suggest that individuals with a balanced TP profile are more effective at dealing with demands of everyday life and adapt better to change.

Although the consequences of having a BTP have been widely studied (e.g., within positive psychology; see Boniwell et al., 2010), its foundations are poorly understood. One of the factors that may contribute to development of a BTP is cognitive functioning. The proponents of the BTP construct defined it as “the mental ability to switch effectively among TPs depending on task features, situational considerations, and personal resources, rather than be biased toward a specific TP that is not adaptive across situations” (Zimbardo and Boyd, 1999, p. 1285). In other words they stated explicitly that BTP is a content-specific type of *mental ability*. Furthermore, it is emphasized that the core feature of BTP is the flexible use of different temporal perspectives, in contrast to the automatic, non-reflective use of externally determined temporal perspectives typical of those with unbalanced TP profiles (Zimbardo and Boyd, 1999, 2008; Stolarski et al., 2016). The descriptions of BTP include some clear references to intellectual ability and imply that cognitive resources might be necessary to use temporal perspectives effectively. Cognitive flexibility enables individuals to achieve their goals and override automatic processes and is usually regarded as a component of executive functioning (also referred to as *executive control*; Miyake et al., 2000; Diamond, 2013; Chuderski and Smolen, 2016). Recent studies have shown that two types of executive function appear to be relevant to personality, namely working memory capacity and response inhibition (Hofmann et al., 2012). In this study we focus on the latter, because it seems especially promising in the research devoted to BTP. Inhibition is the ability to “deliberately inhibit dominant, automatic, or pre-potent responses when necessary” (Miyake et al., 2000, p. 57). Inhibition has been shown to be important for self-regulation, especially for the regulation of emotional reactions. For instance, Tang and Schmeichel (2014) have found that high level of inhibition decreases the intensity of negative emotions. Moreover, Wilkowski and Robinson (2010) have shown that higher cognitive inhibition is associated with lower anger and hostility.

We believe that there are both theoretical and empirical reasons to link inhibition with BTP. At the conceptual level, we refer to the definition of BTP. As we noticed above, Zimbardo and Boyd (1999, 2008) state that BTP helps to be less temporally biased, that is to override automatic reactions rooted in the past, present or future. One may wonder whether people with less balanced TPs also show worse response inhibition i.e., find it more difficult to override dominant (or externally induced) temporal perspectives when they are inappropriate to a particular situation e.g., people biased toward Present-Hedonistic perspective might not be able to activate a Future perspective when it is important to consider the consequences of their actions. Moreover, from the empirical perspective, one can conclude that BTP and inhibition have similar consequences for self-regulation, such as more adaptive emotional responding. These similarities prompt the question of whether and how these seemingly distinct constructs are related. Basing on theoretical analysis and empirical data we hypothesized that BTP would be associated with a more effective inhibition (H1).

Existing evidence suggests that it may be worth considering an additional variable, fluid intelligence, when trying to understand the relationship between executive control and BTP. Fluid intelligence is the ability to reason abstractly and solve novel problems (Cattell, 1971). Empirical data show that executive functions (including inhibition) are strongly associated with fluid intelligence, and consequently some researchers suggested that they might be one of the most important determinants of fluid ability (e.g., Conway et al., 2003). Furthermore, some features of fluid intelligence may promote adaptive activation of the temporal perspective most appropriate to a given situation. In particular, because fluid intelligence is an ability that allows us to adapt our thinking to a new cognitive problem or situation (Carpenter et al., 1990), it may enable effective ‘time horizon management’ (i.e., conscious and intentional adapting one’s own temporal focus in a response to contextual demands) across the varying situations that individuals encounter in everyday life. Fluid intelligence is also considered one of the most important factors in learning (Jaeggi et al., 2008); it may facilitate the drawing of conclusions from one’s experiences and the development of TP-related adaptations, or skills, thus indirectly promoting the development of BTP. The ability to time travel mentally, which is essential to TP-related phenomena, is considered uniquely human (Suddendorf and Corballis, 2007) and may thus be assumed to be linked to high-level cognitive functioning. Importantly, recent studies have provided empirical evidence that BTP is positively associated with fluid intelligence (Zajenkowski et al., 2016). We aimed, therefore, to examine fluid ability as a potential mediator of the relationship between executive control and BTP (H2).

## Materials and Methods

This study, including the consent process, was approved by the ethics committee of Faculty of Psychology at University of Warsaw. Informed consent was obtained from all participants. Participation was voluntary and participants were allowed to reject or withdraw at any point with no disadvantage to their treatments.

### Participants and Procedure

A total of 233 subjects participated in the study (123 women, 110 men). They were students of various universities in Warsaw, Poland recruited via social networking services. The mean age of the sample was 23.55 years (*SD* = 3.70) with a range of 18–39 years. One participant was removed from the sample due to missing data for the cognitive tasks. All participants were tested individually, in a quiet laboratory, in the presence of one experimenter during one session. The task order was one and the same for all subjects. Each experimental session started with the anti-saccade task and then the Go/No-go task. Next, participants completed the Zimbardo Time Perspective Inventory (ZTPI). Finally, their fluid intelligence was assessed with Raven’s Advanced Progressive Matrices (APM) and Cattell’s Culture Fair Intelligence Test (in this order).

### Materials

**Time perspective** was assessed with the ZTPI (Zimbardo and Boyd, 1999). The measure consists of 56 items organized into five scales: Past Negative (e.g., ‘I think about the bad things that have happened to me in the past’), Present Hedonistic (e.g., ‘I try to live my life as fully as possible, one day at a time’), Future (e.g., ‘Meeting tomorrow’s deadlines and doing other necessary work come before tonight’s play’), Past Positive (e.g., ‘It gives me pleasure to think about my past’) and Present Fatalistic (e.g., ‘Fate determines much in my life’). Respondents indicate the extent to which they endorse each item statement using a 5-point Likert scale. We calculated scores for deviation from a balanced time perspective (DBTP; Stolarski et al., 2011), a continuous indicator of the extent to which an individual’s TP profile approximates the optimal TP profile; the lower the DBTP score, the more balanced the individual’s TP profile. Based on their collective cross-cultural database Zimbardo and Boyd (2008) proposed optimal scores for all the TP scales making up the ZTPI.

**Fluid intelligence** was measured with two well-established tests.

*Raven’s Advanced Progressive Matrices* (Raven et al., 1983) is a non-verbal test of abstract reasoning. It consists of 48 items (12 in trial Set I, and 36 in Set II). Participants were presented with different geometric patterns, each missing a piece. Their task was to infer the relationships between the elements and choose one of eight options so as to fill the empty space correctly. The APM score was the sum of all correct choices from Set II. Time limits of 5 and 30 min were imposed for Sets I and II, respectively.

*Cattell’s Culture Fair Intelligence Test* (CFT; Cattell, 1973) consists of four non-verbal subtests with strict time limits. The first part, *Series*, consists of 13 items each comprising a series of three abstract shapes/figures with one piece missing. Respondents must complete the series by selecting the single correct answer from six options. In the subtest *Classifications* respondents are required to identify the two patterns from a set of five which do not belong to the group; there are 14 set of patterns. The *Matrices* subtest is similar to the APM test: only one of six choices fits the blank the blank space in each of 13 matrices. The *Conditions* subtest (10 items) requires the respondent to select one out of five answers in order to replicate the relationships between figures and dot in the model. The total number of correct answers across all subtests constituted the CFT final score.

**Executive control** was assessed with two tasks that require deliberate inhibition of pre-potent, automatic responses by either looking away from the stimulus presented or not responding to certain type of stimulus (Diamond, 2013).

The *anti-saccade task* used was similar to the one described by Chuderski et al. (2012). The procedure was as follows. First, a fixation point appeared at the center of the screen (1500-2500 ms) followed by a rapidly flashing black square on either the right or left side of the screen (200 ms; about 16 cm displacement). Right after that a small arrow pointing downward or to the right or left was presented on the opposite side to the square for 150 ms and then it was replaced with a mask. In the task participants were instructed to make a voluntary eye movement away from the flashing square and then were required to indicate the direction of the arrow by pressing the corresponding key on a keyboard. There were 60 trials and the score was the total number of correct responses.

A *Go/No-go task* similar to the one described by Eimer (1993) was used. Participants were instructed to categorize presented digits (1 to 8) as odd or even. First they performed 60 practice trials to ensure that a strong stimulus-response association was formed, then the experimental condition was introduced. This required them to assign digits 3 to 8 to the aforementioned categories but to inhibit the response to other digits (1 and 2). There were 120 experimental trials (90 go trials; 30 no-go trials). The maximum response latency was 2 s for all trials. Score was the number of inhibited responses to no-go stimuli.

## Results

In the present study we have obtained results from various measures of intelligence and executive control. First we present correlation of all variables. Subsequently, we decided to use structural equation modeling in the main analysis with two latent variables of fluid intelligence and executive control.

**Table 1** presents correlations between TPs, measures of intelligence and cognitive tasks. Most importantly, the results indicate that the DBTP was negatively correlated with both measures of fluid intelligence and performance on the anti-saccade task; it was not associated with performance on the Go/No-go task, although the direction of the correlation was negative. Overall these results indicate that a less balanced TP profile is linked to lower scores on intelligence tests and poorer performance on an executive control task. There were positive correlations between both measures of intelligence and both cognitive tasks.

**Table 1:** Correlations between time perspectives, measures of intelligence and executive control.

	1	2	3	4	5	6	7	8	9	10
(1) Past-negative	–									
(2) Past-positive	0.185 (0.005)	–								
(3) Present-hedonistic	0.209 (0.001)	0.340 (<0.001)	–							
(4) Present-fatalistic	0.415 (<0.001)	0.416 (<0.001)	0.505 (<0.001)	–						
(5) Future	0.051 (0.445)	-0.021 (0.746)	-0.177 (0.007)	-0.166 (0.011)	–					
(6) DBTP	0.530 (<0.001)	-0.337 (<0.001)	-0.075 (0.259)	0.419 (<0.001)	-0.128 (0.053)	–				
(7) Raven	-0.103 (0.119)	-0.099 (0.135)	-0.111 (0.092)	-0.298 (<0.001)	-0.053 (0.419)	-0.193 (0.003)	–			
(8) Cattell	-0.119 (0.071)	-0.079 (0.230)	-0.005 (0.938)	-0.212 (0.001)	-0.003 (0.964)	-0.205 (0.002)	0.686 (<0.001)	–		
(9) Anti-saccade	-0.111 (0.094)	-0.075 (0.259)	-0.052 (0.429)	-0.193 (0.003)	-0.058 (0.385)	-0.132 (0.046)	0.412 (<0.001)	0.455 (<0.001)	–	
(10) Go/No-go	0.041 (0.541)	0.000 (0.997)	-0.024 (0.720)	-0.067 (0.312)	0.078 (0.241)	-0.050 (0.450)	0.139 (0.036)	0.141 (0.033)	0.244 (<0.001)	–
α	0.84	0.66	0.81	0.77	0.80	–	0.90	0.76	0.92	0.90
*M*	2.909	3.161	3.305	2.413	3.417	2.455	22.181	25.017	45.209	25.787
*SD*	0.768	0.496	0.568	0.673	0.469	0.560	6.928	5.006	8.171	4.634

*Exact *p* values are given in brackets. DBTP, Deviation from Balanced Time Perspective.*

**FIGURE 1:**
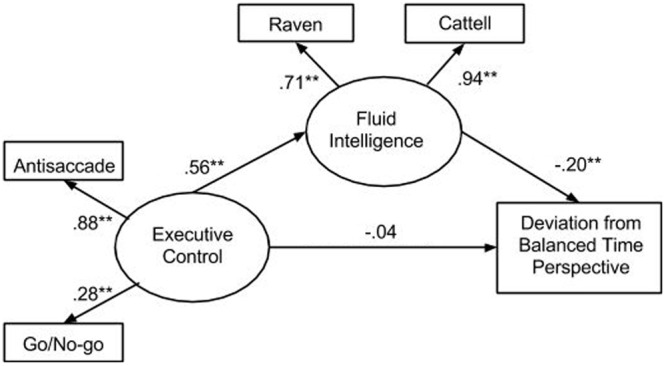
**Model of mediation of the relationship between executive control and deviation from balanced time perspective by fluid intelligence.** ^∗∗^*p* < 0.001.

Next we tested whether the latent variable of fluid intelligence mediated the relationship between executive control (latent variable) and DBTP (**Figure 1**). Four goodness of fit indices were used to evaluate models based on confirmatory factor analysis and structural equation modeling: relative chi-squared (χ^2^/*df*), Bentler’s comparative fit index (CFI), the root mean square of approximation (RMSEA), and Tucker-Lewis Index (TLI). The analysis revealed that our model fitted the data very well (*N* = 232, *df* = 4, χ^2^/df = 0.92, CFI = 1, RMSEA = 0.00, TLI = 1). The path between the latent variable executive control and Deviation from BTP (-0.15, *p* < 0.05), became non-significant when fluid intelligence was included in the analysis as a mediator, which suggested the relationship between executive control and DBTP is fully mediated by fluid intelligence. Subsequently, we have calculated the mediation effect size according to the formula 
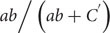
 recommended by Iacobucci et al. (2007) which describes the ratio of the indirect effect to the total effect. In the present analysis the effect size was 0.75.

It has to be acknowledged that in the model presented above, the loading of Go/No-go task on the executive control factor was very low. Thus, we have conducted two additional structural equation modeling (SEM) analyses using the two executive control tasks separately in each model. We have found that, in comparison to our prior analysis, the fit was worse for the SEM with anti-saccade task (*N* = 232, *df* = 2, χ^2^/*df* = 1.74, CFI = 0.99, RMSEA = 0.057, TLI = 0.96). For the SEM with Go/No-go, the indices showed better fit to the data (*N* = 232, *df* = 2, χ^2^/*df* = 0.69, CFI = 1, RMSEA = 0.00, TLI = 1), however, in this case, the total effect (Go/No-go with balanced TP) was small (-0.05).

## Discussion

The BTP concept proposed by Zimbardo and Boyd (1999) implies that individual differences in cognitive functioning underpin TP profile. In this study we examined the contribution of executive control and fluid intelligence to BTP. BTP was shown to be associated with executive control and fluid intelligence, supporting our major predictions. We also found that cognitive ability mediated the relationship between executive control and BTP. These results are consistent with Zimbardo and Boyd’s (1999) theory and suggest that cognitive resources may indeed be important for developing temporal balance.

The magnitude of the correlations was rather small; however, one should bear in mind that these were correlations between performance and self-report indicators and such correlations often underestimate the actual association between constructs (cf. correlations between ability-based and self-report emotional intelligence; Goldenberg et al., 2006). The effects we observed may also have been attenuated by the deficiency of the indicator of TP profile that we used (Stolarski et al., 2015). Although the deviation from BTP seems to be the most valid among hitherto Zimbardo Time Perspective Inentory-based indicators of BTP (Zhang et al., 2013), it is still far from optimal due to (1) the limited reliability of the ZTPI scales, (2) self-report nature of the inventory, and (3) the fact that it measures only a static fit to the ‘optimal’ TP profile and thus represents a disposition rather than a behavioral measure of switching between the various possible TPs. This latter issue is of particular importance, as an adaptive (or optimal) TP profile is merely a precondition for effective management of time horizon in response to situational demands. Moderately high scores for all the ‘positive’ TPs (i.e., Past-Positive, Present-Hedonistic and Future) might indicate that they are relatively accessible to the individual (Zimbardo and Boyd, 2008); however, it does not guarantee that they will be used appropriately. We might have obtained larger effects if we had been able to use a performance-based indicator of the dynamic selection of TPs. Unfortunately, no reliable indicator of this type has yet been developed (Stolarski et al., 2015). Nonetheless the results of this study seem to confirm the assumption that the ‘optimal’ TP profile proposed by Zimbardo and Boyd (2008) may indeed represent a precondition for effective switching between time horizons.

An issue of the currently activated TP consciousness also seems worth considering in the light of the present analyses. Zimbardo and Boyd (1999, p. 1271) described TP as “the often non-conscious process” of activating a particular time horizon. However, they go on to define BTP as the “ability to switch effectively among TPs depending on task features, situational considerations, and personal resources” (p. 1285), suggesting that it represents a more or less conscious form of temporal-self regulation, resulting from reflective use of one’s cognitive resources. One might therefore claim that BTP amounts to being aware of and adapting one’s TP response to situational demands. Following this line of reasoning, and given the cognitive nature of BTP, one might describe BTP as a product of metacognitive self-regulation processes. If one accepts this, it would be particularly valuable to reconsider the BTP concept from the perspective of theories of metacognition (cf. Flavell, 1979).

In this study we measured executive control via tasks engaging inhibition processes, because previous data (e.g., Hofmann et al., 2012) and theoretical analysis led us to think that this function might be especially relevant for BTP. Although there are reasons to link inhibition with BTP, it would be valuable to conduct similar analyses using tasks which measure individual differences in other executive functions such as working memory and attentional shifting. It is possible, although at this stage it is only supposition, that the executive processes of inhibition and shifting relate to different aspects of BTP, for example the former might allow individuals to avoid adopting ‘maladaptive’ TPs (i.e., Past-Negative and Present-Fatalistic), whereas the latter might facilitate effective switching between the remaining ‘positive’ TPs. Therefore, future studies should definitely include measures of cognitive shifting [e.g., similar to those used by Miyake and Friedman (2012)], as attentional flexibility indeed seems crucial for intentional switching between particular time horizons. Shifting processes may in fact prove even more important for BTP than inhibition, as they more directly reflect the process of ‘temporal switching’ that remains the core of BTP definition (Zimbardo and Boyd, 1999). Interaction effects between inhibition and shifting in predicting BTP also seems plausible, as to develop temporal balance one needs to be capable of restraining the ‘maladaptive’ TPs and, at the same time, effectively choose from the ‘positive’ TPs in response to situational demands. Thus, a coincidence of high efficacy in both these executive functions may prove to be optimal for effective temporal balancing. Finally, many studies have emphasized the role of working memory capacity in activating the controlled system (as opposed to the automatic system) that is responsible for conscious, intentional and effortful self-regulation (e.g., Feldman-Barrett et al., 2004). Interestingly, Stolarski and Cyniak-Cieciura (2016) recently showed that there are two temperaments which act as a foundation for BTP: low emotional reactivity and briskness. Low emotional reactivity seems to enable avoidance of uncontrolled activation of maladaptive TPs, whereas briskness appears to facilitate switching between temporal horizons. Briskness is directly associated with performance on an attention switching task (Ledzińska et al., 2013), which provides further reason to extend the design used in this study to include a measure of attentional switching. Further research combining assessment of the temperamental and cognitive underpinnings of BTP in order to illustrate their joint or interactive effects on temporal balance would be particularly interesting.

The current study has several limitations. First, the present research had a cross-sectional character. As a result, any inferences about a causal nature of the analyzed relationships are based solely on theoretical considerations and cannot be empirically verified. Although it is probable that executive functions provide bases for the development of BTP, it is also possible that BTP may influence executive functions (e.g., via regulation of stress states during cognitive task performance; see Zajenkowski et al., 2016). It would be then highly desirable to apply longitudinal design in future studies aiming to determine how these abilities and processes develop in order to establish the direction of influence. The period of early adolescence seems optimal for such analyses, as it remains crucial for the development of both executive functions and complex TP-related processes (cf. Zajenkowski et al., 2015). Furthermore, the measures were presented in the same order to all participants, and therefore the potential order effect was not controlled. Finally, it has to be acknowledged that the executive control tasks used in our study were only weakly correlated, which suggests that they might refer to different constructs. Therefore, future research should examine tasks sharing more variance to capture more homogenous phenomenon.

The results of this study raise the question of what correlates of fluid intelligence, other than response inhibition, might be involved in BTP. As we mentioned above, our research did not include assessment of executive functions such as working memory and attentional shifting which are known to be correlated with intelligence (e.g., Diamond, 2013). Additionally, the pre-potent role of cognitive ability in learning processes seems worth considering in this context (Jaeggi et al., 2008). BTP is a complex human adaptive mechanism which requires constant analysis of one’s current environment in order to adapt one’s TP in response to changes in the situation. Any assessment of the most appropriate TP for a given situation is based on previous experience and the ability to learn from experience was listed as one of the main aspects of general intelligence by Spearman (1923).

## Author Contributions

MZ, MS, JW, and OM designed the study, JW and OM conducted the study. MZ, MS, JW, OM, and PŁ wrote the manuscript and performed analyses.

## Conflict of Interest Statement

The authors declare that the research was conducted in the absence of any commercial or financial relationships that could be construed as a potential conflict of interest.

The reviewers FS and HB and the handling Editor declared their shared affiliation, and the handling Editor states that the process nevertheless met the standards of a fair and objective review.
